# Supplementary material for: Risk of Secondary Cancer after Adjuvant Tamoxifen Treatment for Ductal Carcinoma In Situ: A Nationwide Cohort Study in South Korea
Source: Diagnostics (Basel). 2023 Feb 20;13(4):792. doi: 10.3390/diagnostics13040792 (PMC9954831; doi:10.3390/diagnostics13040792)
Supplement: Supplementary file 1 [file diagnostics-13-00792-s001.zip › diagnostics-2143508-Tables S1-S14.pdf]

Supplementary Table S1. Risk factors of all-site cancer

outcome	Variable		Before matching				After matching			
			Univariable model		Multivariable model		Univariable model		Multivariable model	
			HR(95% CI)	p-value	HR(95% CI)	p-value	HR(95% CI)	p-value	HR(95% CI)	p-value
All-site cancer	Tamoxifen	No	ref		ref		ref		ref	
		Yes	1.014(0.796-1.290)	0.9131	1.001(0.786-1.275)	0.9959	0.953(0.720-1.261)	0.7342	0.957(0.723-1.268)	0.7615
	Type of surgery	BCS	ref		ref		ref		ref	
		Mastectomy	0.821(0.545-1.239)	0.3479	0.806(0.534-1.218)	0.3064	0.901(0.578-1.403)	0.644	0.911(0.587-1.415)	0.679
	DM	No	ref		ref		ref		ref	
		Yes	1.231(0.878-1.727)	0.2284	0.999(0.690-1.447)	0.9973	0.994(0.619-1.595)	0.9787	0.820(0.496-1.354)	0.4372
	HTN	No	ref		ref		ref		ref	
		Yes	1.398(1.077-1.815)	0.0119	1.205(0.885-1.640)	0.2361	1.205(0.868-1.672)	0.2653	1.056(0.725-1.538)	0.7768
	Hyperlipidemia	No	ref		ref		ref		ref	
		Yes	1.240(0.964-1.595)	0.094	1.061(0.801-1.404)	0.6797	1.260(0.927-1.712)	0.1406	1.177(0.843-1.643)	0.3397
	COPD	No	ref		ref		ref		ref	
		Yes	0.143(0.020-1.014)	0.0516	0.117(0.016-0.831)	0.032	0.190(0.027-1.356)	0.0977	0.250(0.050-1.237)	0.0892
	CKD	No	ref		ref		ref		ref	
		Yes	1.263(0.405-3.940)	0.6874	1.025(0.324-3.247)	0.9666	1.256(0.312-5.058)	0.7482	1.372(0.387-4.861)	0.6243
	Liver cirrhosis	No	ref		ref		ref		ref	
		Yes	1.226(0.172-8.733)	0.8389	0.950(0.131-6.901)	0.9592	0.970(0.060-15.683)	0.983	0.914(0.058-14.348)	0.9491
	Heart failure	No	ref		ref		ref		ref	
		Yes	2.117(0.875-5.124)	0.0963	1.883(0.763-4.644)	0.1696	1.233(0.306-4.964)	0.7685	1.493(0.433-5.150)	0.5259
	Age		1.017(1.005-1.028)	0.0035	1.014(1.000-1.027)	0.0456	1.014(1.001-1.027)	0.036	1.015(0.999-1.031)	0.0585

HR, hazard ratio; CI, confidence interval; BCS, breast conserving surgery; DM, diabetes; HTN, hypertension; COPD, chronic obstructive pulmonary disease; CKD, chronic kidney disease

Yellow box highlights the p-value less than 0.05, considered significant.

Supplementary Table S2. Risk factors of thyroid cancer

outcome	Variable		Before matching				After matching			
			Univariable model		Multivariable model		Univariable model		Multivariable model	
			HR(95% CI)	p-value	HR(95% CI)	p-value	HR(95% CI)	p-value	HR(95% CI)	p-value
thyroid	Tamoxifen	No	ref		ref		ref		ref	
		Yes	1.054(0.682-1.628)	0.8128	1.026(0.663-1.587)	0.9081	0.726(0.422-1.251)	0.2488	0.730(0.423-1.257)	0.256
	Type of surgery	wide	ref		ref		ref		ref	
		total	0.505(0.205-1.246)	0.1384	0.562(0.237-1.335)	0.1918	0.567(0.205-1.571)	0.2753	0.653(0.247-1.724)	0.3898
	DM	No	ref		ref		ref		ref	
		Yes	0.964(0.499-1.861)	0.9122	0.965(0.482-1.931)	0.9198	0.539(0.168-1.727)	0.298	0.617(0.202-1.888)	0.3973
	HTN	No	ref		ref		ref		ref	
		Yes	0.995(0.599-1.654)	0.9857	0.952(0.531-1.707)	0.8691	0.761(0.372-1.556)	0.4538	0.723(0.328-1.590)	0.4192
	Hyperlipidemia	No	ref		ref		ref		ref	
		Yes	1.418(0.915-2.197)	0.1185	1.603(0.986-2.607)	0.0568	1.465(0.832-2.582)	0.1862	1.798(0.976-3.312)	0.0598
	COPD	No	ref		ref		ref		ref	
		Yes	0.225(0.014-3.690)	0.2962	0.211(0.014-3.251)	0.265	0.343(0.021-5.691)	0.4551	0.355(0.024-5.210)	0.4498
	CKD	No	ref		ref		ref		ref	
		Yes	1.281(0.178-9.191)	0.8058	1.918(0.385-9.552)	0.4266	2.253(0.311-16.292)	0.4212	4.099(0.759-22.136)	0.1011
	Liver cirrhosis	No	ref		ref		ref		ref	
		Yes	1.827(0.112-29.940)	0.6726	1.122(0.074-17.008)	0.934	3.417(0.206-56.665)	0.3911	2.597(0.164-41.108)	0.4982
	Heart failure	No	ref		ref		ref		ref	
		Yes	4.102(1.297-12.975)	0.0163	6.008(2.055-17.562)	0.0011	4.661(1.135-19.133)	0.0327	7.967(2.218-28.618)	0.0015
	Age		0.993(0.973-1.014)	0.5358	0.988(0.965-1.012)	0.3314	0.991(0.966-1.017)	0.4867	0.992(0.963-1.022)	0.5937

HR, hazard ratio; CI, confidence interval; BCS, breast conserving surgery; DM, diabetes; HTN, hypertension; COPD, chronic obstructive pulmonary disease; CKD, chronic kidney disease

Yellow box highlights the p-value less than 0.05, considered significant.

Supplementary Table S3. Risk factors of ovarian cancer

outcome	Variable		Before matching				After matching			
			Univariable model		Multivariable model		Univariable model		Multivariable model	
			HR(95% CI)	p-value	HR(95% CI)	p-value	HR(95% CI)	p-value	HR(95% CI)	p-value
ovary	Tamoxifen	No	ref		ref		ref		ref	
		Yes	1.059(0.570-1.969)	0.8562	1.049(0.564-1.950)	0.8807	1.004(0.484-2.083)	0.9918	0.999(0.481-2.074)	0.998
	Type of surgery	wide	ref		ref		ref		ref	
		total	0.885(0.316-2.476)	0.8156	0.971(0.362-2.600)	0.9529	1.157(0.402-3.324)	0.787	1.361(0.493-3.751)	0.5518
	DM	No	ref		ref		ref		ref	
		Yes	0.796(0.284-2.228)	0.6639	0.813(0.290-2.275)	0.6927	0.333(0.045-2.445)	0.2795	0.607(0.120-3.060)	0.545
	HTN	No	ref		ref		ref		ref	
		Yes	1.567(0.817-3.003)	0.1763	2.047(0.952-4.403)	0.0667	0.791(0.302-2.074)	0.6339	1.215(0.433-3.408)	0.7118
	Hyperlipidemia	No	ref		ref		ref		ref	
		Yes	1.061(0.543-2.070)	0.8632	1.067(0.512-2.225)	0.8629	0.944(0.402-2.212)	0.8938	1.291(0.527-3.162)	0.5759
	COPD	No	ref		ref		ref		ref	
		Yes	0.485(0.029-8.147)	0.615	0.474(0.032-7.062)	0.5881	0.646(0.038-11.105)	0.7635	0.770(0.055-10.718)	0.8459
	CKD	No	ref		ref		ref		ref	
		Yes	2.931(0.403-21.321)	0.2883	3.561(0.681-18.626)	0.1324	2.085(0.121-35.899)	0.6128	2.252(0.126-40.203)	0.5809
	Liver cirrhosis	No	ref		ref		ref		ref	
		Yes	3.992(0.237-67.220)	0.3366	2.514(0.168-37.698)	0.5046	6.552(0.380-112.958)	0.1957	7.918(0.542-115.620)	0.1304
	Heart failure	No	ref		ref		ref		ref	
		Yes	2.633(0.362-19.136)	0.3386	4.007(0.814-19.738)	0.0879	1.876(0.109-32.251)	0.6646	2.230(0.166-30.052)	0.5455
	Age		0.988(0.959-1.018)	0.4386	0.978(0.945-1.012)	0.2023	0.976(0.941-1.012)	0.1868	0.978(0.939-1.019)	0.2969

HR, hazard ratio; CI, confidence interval; BCS, breast conserving surgery; DM, diabetes; HTN, hypertension; COPD, chronic obstructive pulmonary disease; CKD, chronic kidney disease

Yellow box highlights the p-value less than 0.05, considered significant.

Supplementary Table S4. Risk factors of melanoma

outcome	Variable		Before matching				After matching			
			Univariable model		Multivariable model		Univariable model		Multivariable model	
			HR(95% CI)	p-value	HR(95% CI)	p-value	HR(95% CI)	p-value	HR(95% CI)	p-value
melanoma	Tamoxifen	No	ref		ref		ref		ref	
		Yes	0.409(0.068-2.458)	0.3287	0.463(0.070-3.073)	0.4249	0.723(0.120-4.344)	0.7229	0.779(0.122-4.983)	0.7923
	Type of surgery	wide	ref		ref		ref		ref	
		total	2.159(0.241-19.325)	0.4913	2.718(0.336-21.984)	0.3484	1.812(0.202-16.218)	0.5952	2.608(0.318-21.411)	0.3721
	DM	No	ref		ref		ref		ref	
		Yes	1.926(0.215-17.266)	0.558	5.903(0.562-61.943)	0.1388	2.364(0.264-21.190)	0.442	6.354(0.578-69.896)	0.1307
	HTN	No	ref		ref		ref		ref	
		Yes	0.329(0.014-7.856)	0.4923	0.264(0.017-4.151)	0.3434	0.349(0.015-8.327)	0.5153	0.205(0.008-5.221)	0.3371
	Hyperlipidemia	No	ref		ref		ref		ref	
		Yes	0.243(0.010-5.843)	0.3834	0.215(0.012-3.865)	0.2968	0.272(0.011-6.537)	0.4224	0.292(0.020-4.352)	0.3716
	COPD	No	ref		ref		ref		ref	
		Yes	3.832(0.159-92.413)	0.4081	2.335(0.076-71.480)	0.6272	3.531(0.147-84.613)	0.4364	1.478(0.036-60.278)	0.8365
	CKD	No	ref		ref		ref		ref	
		Yes	10.747(0.443-260.849)	0.1445	10.322(0.406-262.444)	0.1574	11.403(0.474-274.558)	0.1337	12.089(0.372-392.574)	0.1605
	Liver cirrhosis	No	ref		ref		ref		ref	
		Yes	31.559(1.290-771.791)	0.0343	16.043(0.334-769.491)	0.1599	37.176(1.505-918.122)	0.0271	6.583(0.049-875.782)	0.4501
	Heart failure	No	ref		ref		ref		ref	
		Yes	10.501(0.438-251.492)	0.1467	4.348(0.112-168.093)	0.4306	10.497(0.440-250.618)	0.1464	21.454(0.468-984.069)	0.1163
	Age		0.988(0.904-1.079)	0.7848	1.013(0.926-1.109)	0.7781	0.992(0.911-1.079)	0.8441	1.012(0.928-1.103)	0.7856

HR, hazard ratio; CI, confidence interval; BCS, breast conserving surgery; DM, diabetes; HTN, hypertension; COPD, chronic obstructive pulmonary disease; CKD, chronic kidney disease

Yellow box highlights the p-value less than 0.05, considered significant.

Supplementary Table S5. Risk factors of thoracic cancer*

outcome	Variable		Before matching				After matching			
			Univariable model		Multivariable model		Univariable model		Multivariable model	
			HR(95% CI)	p-value	HR(95% CI)	p-value	HR(95% CI)	p-value	HR(95% CI)	p-value
thorax	Tamoxifen	No	ref		ref					
		Yes	1.897(0.020-184.083)	0.7838	2.777(0.015-513.123)	0.7013				
	Type of surgery	wide	ref		ref					
		total	2.843(0.030-265.184)	0.6516	2.650(0.011-623.442)	0.7264				
	DM	No	ref		ref					
		Yes	2.446(0.026-226.868)	0.6987	0.105(0.000-50.012)	0.4732				
	HTN	No	ref		ref					
		Yes	10.700(0.115-994.421)	0.3053	1.986(0.009-460.779)	0.8051				
	Hyperlipidemia	No	ref		ref					
		Yes	7.931(0.085-736.887)	0.3705	5.109(0.025-1038.337)	0.5475				
	COPD	No	ref		ref					
		Yes	13.062(0.141-1214.214)	0.2665	5.830(0.013-2535.920)	0.5695				
	CKD	No	ref		ref					
		Yes	36.445(0.397-3348.581)	0.119	25.630(0.064-10344.24)	0.2894				
	Liver cirrhosis	No	ref		ref					
		Yes	99.915(1.061-9411.537)	0.0471	27.876(0.011-73899.20)	0.408				
	Heart failure	No			ref					
		Yes			146.764(0.927-23245.50)	0.0535				
	Age		1.103(0.931-1.307)	0.2556	1.026(0.820-1.285)	0.8204				

HR, hazard ratio; CI, confidence interval; BCS, breast conserving surgery; DM, diabetes; HTN, hypertension; COPD, chronic obstructive pulmonary disease; CKD, chronic kidney disease

Yellow box highlights the p-value less than 0.05, considered significant.

*The number of events were too small, matched analysis was not conducted.

Supplementary Table S6. Risk factors of gastrointestinal cancer

outcome	Variable		Before matching				After matching			
			Univariable model		Multivariable model		Univariable model		Multivariable model	
			HR(95% CI)	p-value	HR(95% CI)	p-value	HR(95% CI)	p-value	HR(95% CI)	p-value
all gastrointestianl	Tamoxifen	No	ref		ref		ref		ref	
		Yes	0.887(0.551-1.429)	0.6232	0.877(0.544-1.414)	0.5899	0.940(0.545-1.624)	0.8258	0.939(0.544-1.623)	0.8224
	Type of surgery	wide	ref		ref		ref		ref	
		total	1.249(0.620-2.515)	0.5336	1.249(0.627-2.491)	0.5269	1.300(0.612-2.762)	0.4946	1.295(0.617-2.718)	0.4941
	DM	No	ref		ref		ref		ref	
		Yes	1.599(0.858-2.979)	0.1391	1.227(0.628-2.400)	0.5494	1.216(0.519-2.848)	0.6526	0.859(0.357-2.064)	0.7334
	HTN	No	ref		ref		ref		ref	
		Yes	1.672(1.010-2.770)	0.0459	1.303(0.717-2.369)	0.3857	1.557(0.855-2.837)	0.148	1.200(0.600-2.400)	0.6064
	Hyperlipidemia	No	ref		ref		ref		ref	
		Yes	1.164(0.697-1.943)	0.5625	0.821(0.465-1.448)	0.4953	1.347(0.746-2.430)	0.3231	1.055(0.556-2.004)	0.8695
	COPD	No	ref		ref		ref		ref	
		Yes	0.598(0.083-4.304)	0.6093	0.652(0.130-3.277)	0.6036	0.753(0.104-5.444)	0.7783	0.827(0.163-4.195)	0.819
	CKD	No	ref		ref		ref		ref	
		Yes	0.814(0.049-13.411)	0.8853	0.444(0.028-7.021)	0.5642	1.154(0.069-19.210)	0.9207	0.838(0.050-14.183)	0.9027
	Liver cirrhosis	No	ref		ref		ref		ref	
		Yes	5.137(0.713-37.024)	0.1044	9.166(1.907-44.059)	0.0057	3.751(0.224-62.665)	0.3575	4.082(0.270-61.697)	0.31
	Heart failure	No	ref		ref		ref		ref	
		Yes	0.787(0.048-12.963)	0.8668	0.388(0.026-5.885)	0.4947	1.070(0.064-17.819)	0.9625	0.736(0.052-10.388)	0.8207
	Age		1.034(1.012-1.057)	0.002	1.033(1.007-1.060)	0.0133	1.033(1.008-1.059)	0.0087	1.034(1.005-1.065)	0.0236

HR, hazard ratio; CI, confidence interval; BCS, breast conserving surgery; DM, diabetes; HTN, hypertension; COPD, chronic obstructive pulmonary disease; CKD, chronic kidney disease

Yellow box highlights the p-value less than 0.05, considered significant.

Supplementary Table S7. Risk factors of gastric cancer

outcome	Variable		Before matching				After matching			
			Univariable model		Multivariable model		Univariable model		Multivariable model	
			HR(95% CI)	p-value	HR(95% CI)	p-value	HR(95% CI)	p-value	HR(95% CI)	p-value
gastric	Tamoxifen	No	ref		ref		ref		ref	
		Yes	0.762(0.309-1.880)	0.5549	0.753(0.303-1.871)	0.5407	0.652(0.218-1.950)	0.4437	0.656(0.217-1.978)	0.4537
	Type of surgery	wide	ref		ref		ref		ref	
		total	1.022(0.236-4.424)	0.9772	1.036(0.267-4.026)	0.9588	1.217(0.272-5.437)	0.7974	1.291(0.315-5.301)	0.7228
	DM	No	ref		ref		ref		ref	
		Yes	0.962(0.222-4.167)	0.9587	0.626(0.158-2.477)	0.505	0.748(0.098-5.721)	0.7798	0.505(0.088-2.898)	0.4434
	HTN	No	ref		ref		ref		ref	
		Yes	2.162(0.851-5.491)	0.1051	1.587(0.541-4.653)	0.4005	2.165(0.726-6.462)	0.1661	1.672(0.482-5.801)	0.418
	Hyperlipidemia	No	ref		ref		ref		ref	
		Yes	0.797(0.264-2.411)	0.6884	0.494(0.159-1.536)	0.2228	0.889(0.247-3.195)	0.8564	0.570(0.156-2.083)	0.3953
	COPD	No	ref		ref		ref		ref	
		Yes	2.510(0.334-18.836)	0.3709	2.457(0.442-13.650)	0.3044	3.110(0.406-23.793)	0.2745	3.039(0.533-17.330)	0.2109
	CKD	No	ref		ref		ref		ref	
		Yes	3.343(0.186-60.007)	0.4127	2.244(0.137-36.905)	0.5714	4.606(0.247-85.846)	0.3061	3.542(0.177-70.955)	0.4083
	Liver cirrhosis	No	ref		ref		ref		ref	
		Yes	9.511(0.524-172.536)	0.1277	9.769(0.617-154.716)	0.1059	15.566(0.816-296.791)	0.068	17.616(0.817-379.873)	0.0671
	Heart failure	No	ref		ref		ref		ref	
		Yes	3.019(0.169-53.896)	0.4523	1.153(0.070-18.877)	0.9205	4.083(0.220-75.727)	0.3451	1.485(0.082-26.792)	0.7889
	Age		1.060(1.019-1.102)	0.0038	1.068(1.020-1.117)	0.0046	1.054(1.007-1.103)	0.0242	1.061(1.007-1.118)	0.0267

HR, hazard ratio; CI, confidence interval; BCS, breast conserving surgery; DM, diabetes; HTN, hypertension; COPD, chronic obstructive pulmonary disease; CKD, chronic kidney disease

Yellow box highlights the p-value less than 0.05, considered significant.

Supplementary Table S8. Risk factors of colorectal cancer

outcome	Variable		Before matching				After matching			
			Univariable model		Multivariable model		Univariable model		Multivariable model	
			HR(95% CI)	p-value	HR(95% CI)	p-value	HR(95% CI)	p-value	HR(95% CI)	p-value
colorectal	Tamoxifen	No	ref		ref		ref		ref	
		Yes	1.451(0.511-4.123)	0.485	1.475(0.527-4.126)	0.4592	1.470(0.466-4.637)	0.5109	1.405(0.445-4.436)	0.5625
	Type of surgery	wide	ref		ref		ref		ref	
		total	2.569(0.838-7.877)	0.0989	2.699(0.897-8.121)	0.0772	2.342(0.634-8.649)	0.2019	2.513(0.690-9.147)	0.1622
	DM	No	ref		ref		ref		ref	
		Yes	1.000(0.229-4.373)	>.9999	1.619(0.395-6.640)	0.5035	0.825(0.106-6.388)	0.8536	1.299(0.217-7.788)	0.775
	HTN	No	ref		ref		ref		ref	
		Yes	0.774(0.222-2.694)	0.6874	1.102(0.298-4.073)	0.8847	0.761(0.167-3.474)	0.7247	1.052(0.224-4.939)	0.9486
	Hyperlipidemia	No	ref		ref		ref		ref	
		Yes	0.074(0.004-1.332)	0.0775	0.051(0.004-0.682)	0.0244	0.115(0.006-2.176)	0.1492	0.079(0.005-1.149)	0.0631
	COPD	No	ref		ref		ref		ref	
		Yes	1.134(0.063-20.485)	0.9322	0.966(0.066-14.086)	0.9796	1.482(0.078-28.081)	0.7934	1.010(0.060-17.058)	0.9943
	CKD	No	ref		ref		ref		ref	
		Yes	3.151(0.175-56.896)	0.4368	4.787(0.296-77.518)	0.2704	4.618(0.243-87.665)	0.3083	6.584(0.328-132.006)	0.218
	Liver cirrhosis	No	ref		ref		ref		ref	
		Yes	8.852(0.490-160.063)	0.1398	12.661(0.859-186.529)	0.0644	14.585(0.768-277.116)	0.0744	16.289(0.851-311.613)	0.0639
	Heart failure	No	ref		ref		ref		ref	
		Yes	3.218(0.178-58.101)	0.4286	2.464(0.154-39.428)	0.5239	4.670(0.246-88.769)	0.3051	3.292(0.168-64.638)	0.4328
	Age		1.013(0.968-1.060)	0.5745	1.037(0.987-1.090)	0.1486	1.021(0.969-1.075)	0.4404	1.047(0.989-1.108)	0.1134

HR, hazard ratio; CI, confidence interval; BCS, breast conserving surgery; DM, diabetes; HTN, hypertension; COPD, chronic obstructive pulmonary disease; CKD, chronic kidney disease

Yellow box highlights the p-value less than 0.05, considered significant.

Supplementary Table S9. Risk factors of bladder cancer

outcome	Variable		Before matching				After matching			
			Univariable model		Multivariable model		Univariable model		Multivariable model	
			HR(95% CI)	p-value	HR(95% CI)	p-value	HR(95% CI)	p-value	HR(95% CI)	p-value
bladder	Tamoxifen	No	ref		ref		ref		ref	
		Yes	0.664(0.133-3.313)	0.618	0.703(0.132-3.753)	0.6805	0.807(0.134-4.865)	0.8147	0.925(0.140-6.129)	0.9358
	Type of surgery	wide	ref		ref		ref		ref	
		total	0.682(0.030-15.276)	0.8091	0.547(0.028-10.753)	0.6916	0.692(0.029-16.540)	0.82	0.511(0.022-12.135)	0.6781
	DM	No	ref		ref		ref		ref	
		Yes	0.625(0.028-14.058)	0.7672	0.279(0.017-4.579)	0.3711	0.913(0.038-21.971)	0.9555	0.249(0.010-5.905)	0.3894
	HTN	No	ref		ref		ref		ref	
		Yes	0.725(0.085-6.204)	0.769	0.554(0.074-4.174)	0.5669	0.954(0.107-8.536)	0.9667	0.630(0.075-5.280)	0.67
	Hyperlipidemia	No	ref		ref		ref		ref	
		Yes	0.591(0.068-5.096)	0.6319	0.573(0.081-4.062)	0.5773	0.849(0.094-7.660)	0.8841	0.712(0.092-5.508)	0.7449
	COPD	No	ref		ref		ref		ref	
		Yes	3.556(0.156-81.043)	0.4264	2.804(0.131-60.226)	0.51	4.000(0.165-96.944)	0.3941	2.548(0.101-64.351)	0.5702
	CKD	No	ref		ref		ref		ref	
		Yes	9.616(0.423-218.462)	0.1555	10.593(0.466-240.632)	0.1386	12.296(0.510-296.657)	0.1224	11.942(0.366-389.319)	0.163
	Liver cirrhosis	No	ref		ref		ref		ref	
		Yes	28.512(1.235-658.368)	0.0365	25.087(0.900-699.413)	0.0577	39.010(1.571-968.890)	0.0254	45.291(1.049-1956.075)	0.0472
	Heart failure	No	ref		ref		ref		ref	
		Yes	8.357(0.371-188.057)	0.1814	4.370(0.183-104.286)	0.3622	9.573(0.396-231.363)	0.1645	3.194(0.086-118.987)	0.5293
	Age		1.059(0.988-1.135)	0.1039	1.099(1.016-1.190)	0.019	1.071(0.995-1.153)	0.0677	1.109(1.014-1.212)	0.0228

HR, hazard ratio; CI, confidence interval; BCS, breast conserving surgery; DM, diabetes; HTN, hypertension; COPD, chronic obstructive pulmonary disease; CKD, chronic kidney disease

Yellow box highlights the p-value less than 0.05, considered significant.

Supplementary Table S10. Risk factors of head and neck cancer

outcome	Variable		Before matching				After matching			
			Univariable model		Multivariable model		Univariable model		Multivariable model	
			HR(95% CI)	p-value	HR(95% CI)	p-value	HR(95% CI)	p-value	HR(95% CI)	p-value
head and neck	Tamoxifen	No	ref		ref		ref		ref	
		Yes	3.768(0.091-155.351)	0.4845	3.668(0.109-123.552)	0.4689	3.840(0.042-349.623)	0.5589	4.942(0.033-744.797)	0.5324
	Type of surgery	wide	ref		ref		ref		ref	
		total	1.688(0.041-69.541)	0.7826	1.379(0.030-64.084)	0.8697	2.410(0.026-223.320)	0.7035	1.392(0.011-180.850)	0.8941
	DM	No	ref		ref		ref		ref	
		Yes	1.762(0.043-72.600)	0.7652	0.352(0.005-24.659)	0.6299	3.356(0.036-315.133)	0.6013	0.347(0.002-75.059)	0.6995
	HTN	No	ref		ref		ref		ref	
		Yes	3.769(0.236-60.253)	0.3482	1.680(0.065-43.615)	0.7549	12.296(0.132-1148.960)	0.2784	2.664(0.019-365.184)	0.6963
	Hyperlipidemia	No	ref		ref		ref		ref	
		Yes	3.312(0.207-52.954)	0.3971	2.101(0.107-41.207)	0.6248	10.626(0.114-988.466)	0.3068	4.928(0.048-511.074)	0.5006
	COPD	No	ref		ref		ref		ref	
		Yes	9.160(0.222-377.486)	0.2431	6.466(0.072-583.572)	0.4165	12.864(0.138-1197.210)	0.2694	7.269(0.017-3021.481)	0.5191
	CKD	No	ref		ref		ref		ref	
		Yes	30.604(0.741-1263.181)	0.0715	23.843(0.213-2665.167)	0.1875	44.628(0.488-4083.940)	0.0993	22.799(0.046-11388.61)	0.324
	Liver cirrhosis	No	ref		ref		ref		ref	
		Yes	119.992(2.851-5049.957)	0.0121	6.889(0.006-7595.991)	0.5892	206.684(2.224-19204.77)	0.0211	192.741(0.035-1066198)	0.2315
	Heart failure	No	ref		ref		ref		ref	
		Yes	28.638(0.694-1181.075)	0.0771	25.828(0.294-2268.382)	0.1545	48.188(0.528-4399.347)	0.0925	36.974(0.078-17577.62)	0.251
	Age		1.058(0.937-1.195)	0.3594	1.064(0.924-1.227)	0.3887	1.154(0.971-1.372)	0.1035	1.118(0.896-1.394)	0.3239

HR, hazard ratio; CI, confidence interval; BCS, breast conserving surgery; DM, diabetes; HTN, hypertension; COPD, chronic obstructive pulmonary disease; CKD, chronic kidney disease

Yellow box highlights the p-value less than 0.05, considered significant.

Supplementary Table S11. Risk factors of lung cancer

outcome	Variable		Before matching				After matching			
			Univariable model		Multivariable model		Univariable model		Multivariable model	
			HR(95% CI)	p-value	HR(95% CI)	p-value	HR(95% CI)	p-value	HR(95% CI)	p-value
lung	Tamoxifen	No	ref		ref		ref		ref	
		Yes	0.991(0.496-1.983)	0.9806	0.975(0.484-1.962)	0.9425	1.088(0.503-2.353)	0.8295	1.098(0.506-2.385)	0.813
	Type of surgery	wide	ref		ref		ref		ref	
		total	1.139(0.401-3.234)	0.8067	1.197(0.441-3.252)	0.724	0.944(0.283-3.146)	0.9257	1.067(0.342-3.327)	0.9116
	DM	No	ref		ref		ref		ref	
		Yes	2.333(1.056-5.156)	0.0362	1.771(0.740-4.235)	0.1991	1.674(0.577-4.861)	0.3431	1.759(0.575-5.386)	0.3225
	HTN	No	ref		ref		ref		ref	
		Yes	1.305(0.609-2.796)	0.4935	0.676(0.281-1.629)	0.3833	0.501(0.150-1.668)	0.26	0.343(0.102-1.159)	0.085
	Hyperlipidemia	No	ref		ref		ref		ref	
		Yes	1.458(0.720-2.952)	0.2947	1.045(0.484-2.254)	0.9108	0.898(0.360-2.239)	0.8167	0.837(0.327-2.140)	0.7106
	COPD	No	ref		ref		ref		ref	
		Yes	0.587(0.035-9.952)	0.7123	0.331(0.021-5.276)	0.4337	0.713(0.041-12.362)	0.8164	0.613(0.042-9.032)	0.7211
	CKD	No	ref		ref		ref		ref	
		Yes	1.604(0.094-27.281)	0.744	0.971(0.064-14.714)	0.9831	2.180(0.125-37.875)	0.5927	1.748(0.092-33.126)	0.7099
	Liver cirrhosis	No	ref		ref		ref		ref	
		Yes	4.702(0.276-80.187)	0.2848	2.866(0.175-46.928)	0.4605	7.272(0.417-126.816)	0.1738	7.576(0.468-122.691)	0.1541
	Heart failure	No	ref		ref		ref		ref	
		Yes	3.307(0.452-24.195)	0.2388	3.559(0.701-18.073)	0.1257	2.036(0.116-35.583)	0.6263	2.166(0.143-32.726)	0.577
	Age		1.047(1.016-1.079)	0.0026	1.049(1.012-1.088)	0.0083	1.030(0.995-1.066)	0.0956	1.049(1.007-1.092)	0.021

HR, hazard ratio; CI, confidence interval; BCS, breast conserving surgery; DM, diabetes; HTN, hypertension; COPD, chronic obstructive pulmonary disease; CKD, chronic kidney disease

Yellow box highlights the p-value less than 0.05, considered significant.

Supplementary Table S12. Risk factors of leukemia

outcome	Variable		Before matching				After matching			
			Univariable model		Multivariable model		Univariable model		Multivariable model	
			HR(95% CI)	p-value	HR(95% CI)	p-value	HR(95% CI)	p-value	HR(95% CI)	p-value
leukemia	Tamoxifen	No	ref		ref		ref		ref	
		Yes	1.623(0.145-18.159)	0.6945	1.535(0.127-18.597)	0.7363	1.737(0.108-27.935)	0.6969	1.492(0.064-34.551)	0.8031
	Type of surgery	wide	ref		ref		ref		ref	
		total	1.402(0.045-43.279)	0.847	1.260(0.041-38.720)	0.8947	1.882(0.045-78.151)	0.7394	1.366(0.024-76.452)	0.8794
	DM	No	ref		ref		ref		ref	
		Yes	1.318(0.042-40.943)	0.8748	0.768(0.016-37.915)	0.8942	2.480(0.060-102.400)	0.6324	2.377(0.016-347.461)	0.7335
	HTN	No	ref		ref		ref		ref	
		Yes	0.548(0.018-16.817)	0.7309	0.502(0.022-11.474)	0.6659	0.887(0.021-36.690)	0.9497	0.918(0.024-34.833)	0.9634
	Hyperlipidemia	No	ref		ref		ref		ref	
		Yes	0.524(0.016-16.796)	0.7146	0.709(0.030-16.600)	0.8308	0.999(0.024-41.236)	0.9996	1.401(0.033-58.778)	0.8595
	COPD	No	ref		ref		ref		ref	
		Yes	7.241(0.231-226.852)	0.26	5.974(0.128-278.698)	0.3619	9.333(0.223-390.384)	0.241	8.312(0.072-959.150)	0.382
	CKD	No	ref		ref		ref		ref	
		Yes	18.456(0.578-589.335)	0.099	30.714(0.486-1942.436)	0.1055	23.471(0.499-1105.018)	0.1083	26.428(0.118-5930.250)	0.2358
	Liver cirrhosis	No	ref		ref		ref		ref	
		Yes	91.578(1.937-4329.566)	0.0217	26.657(0.167-4256.072)	0.2047	227.450(2.450-21113.50)	0.0189	91.020(0.081-102607.0)	0.2083
	Heart failure	No	ref		ref		ref		ref	
		Yes	12.831(0.414-397.935)	0.1453	19.012(0.472-766.009)	0.1184	13.108(0.310-555.062)	0.1782	15.576(0.162-1499.680)	0.2387
	Age		0.969(0.858-1.094)	0.6066	1.015(0.903-1.140)	0.8075	0.942(0.806-1.100)	0.4509	0.983(0.842-1.148)	0.8277

HR, hazard ratio; CI, confidence interval; BCS, breast conserving surgery; DM, diabetes; HTN, hypertension; COPD, chronic obstructive pulmonary disease; CKD, chronic kidney disease

Yellow box highlights the p-value less than 0.05, considered significant.

Supplementary Table S13. Risk factors of non-Hodgkin lymphoma

outcome	Variable		Before matching				After matching			
			Univariable model		Multivariable model		Univariable model		Multivariable model	
			HR(95% CI)	p-value	HR(95% CI)	p-value	HR(95% CI)	p-value	HR(95% CI)	p-value
non-Hodgkin lymphoma	Tamoxifen	No	ref		ref		ref		ref	
		Yes	0.934(0.208-4.196)	0.9287	0.900(0.195-4.150)	0.892	0.403(0.042-3.897)	0.4322	0.492(0.051-4.801)	0.5421
	Type of surgery	wide	ref		ref		ref		ref	
		total	0.588(0.028-12.544)	0.7336	0.496(0.024-10.093)	0.6484	0.827(0.032-21.680)	0.9091	0.709(0.026-19.555)	0.8392
	DM	No	ref		ref		ref		ref	
		Yes	1.401(0.168-11.665)	0.755	1.019(0.144-7.194)	0.9849	3.364(0.349-32.453)	0.2941	1.114(0.102-12.188)	0.9298
	HTN	No	ref		ref		ref		ref	
		Yes	2.752(0.616-12.299)	0.185	2.115(0.367-12.198)	0.402	11.752(1.222-113.007)	0.0329	5.261(0.438-63.255)	0.1907
	Hyperlipidemia	No	ref		ref		ref		ref	
		Yes	0.514(0.061-4.293)	0.5386	0.312(0.043-2.288)	0.2521	1.113(0.115-10.773)	0.9264	0.403(0.039-4.146)	0.4451
	COPD	No	ref		ref		ref		ref	
		Yes	3.174(0.147-68.752)	0.4617	1.836(0.093-36.139)	0.6893	4.558(0.172-120.644)	0.3641	2.110(0.080-55.561)	0.6546
	CKD	No	ref		ref		ref		ref	
		Yes	8.943(0.413-193.699)	0.1626	4.393(0.175-110.024)	0.3678	15.735(0.591-419.114)	0.0998	5.750(0.131-252.655)	0.3648
	Liver cirrhosis	No	ref		ref		ref		ref	
		Yes	25.678(1.164-566.705)	0.0398	21.126(0.794-562.023)	0.0684	59.073(2.010-1736.340)	0.018	38.143(0.302-4815.601)	0.1402
	Heart failure	No	ref		ref		ref		ref	
		Yes	7.485(0.349-160.601)	0.1982	1.935(0.074-50.685)	0.6919	12.801(0.487-336.586)	0.1264	0.877(0.013-57.828)	0.9512
	Age		1.067(1.001-1.137)	0.048	1.074(0.997-1.157)	0.0586	1.107(1.019-1.203)	0.0158	1.091(0.987-1.206)	0.0874

HR, hazard ratio; CI, confidence interval; BCS, breast conserving surgery; DM, diabetes; HTN, hypertension; COPD, chronic obstructive pulmonary disease; CKD, chronic kidney disease

Yellow box highlights the p-value less than 0.05, considered significant.

Supplementary Table S14. Risk factors of other cancer

outcome	Variable		Before matching				After matching			
			Univariable model		Multivariable model		Univariable model		Multivariable model	
			HR(95% CI)	p-value	HR(95% CI)	p-value	HR(95% CI)	p-value	HR(95% CI)	p-value
Other	Tamoxifen	No	ref		ref		ref		ref	
		Yes	1.510(0.789-2.889)	0.2136	1.461(0.764-2.792)	0.2515	1.388(0.666-2.890)	0.3817	1.373(0.658-2.863)	0.3986
	Type of surgery	wide	ref		ref		ref		ref	
		total	0.406(0.098-1.678)	0.2131	0.479(0.133-1.720)	0.2589	0.537(0.128-2.258)	0.3961	0.643(0.174-2.376)	0.5076
	DM	No	ref		ref		ref		ref	
		Yes	1.227(0.518-2.904)	0.6419	0.910(0.370-2.234)	0.8362	1.521(0.529-4.374)	0.4361	1.123(0.375-3.365)	0.8363
	HTN	No	ref		ref		ref		ref	
		Yes	1.882(1.009-3.511)	0.0467	1.657(0.790-3.475)	0.1811	2.368(1.118-5.013)	0.0243	2.242(0.921-5.455)	0.0751
	Hyperlipidemia	No	ref		ref		ref		ref	
		Yes	1.150(0.601-2.202)	0.6731	0.838(0.411-1.708)	0.6262	1.398(0.635-3.076)	0.405	1.048(0.445-2.467)	0.9143
	COPD	No	ref		ref		ref		ref	
		Yes	0.469(0.028-7.876)	0.5991	0.361(0.023-5.604)	0.4668	0.658(0.038-11.315)	0.7731	0.544(0.038-7.849)	0.6548
	CKD	No	ref		ref		ref		ref	
		Yes	2.776(0.382-20.175)	0.313	3.470(0.671-17.948)	0.1378	4.678(0.636-34.394)	0.1295	4.334(0.760-24.722)	0.0988
	Liver cirrhosis	No	ref		ref		ref		ref	
		Yes	3.886(0.231-65.351)	0.3459	2.243(0.144-35.018)	0.5645	7.204(0.415-125.001)	0.175	7.162(0.462-110.936)	0.1591
	Heart failure	No	ref		ref		ref		ref	
		Yes	2.603(0.358-18.908)	0.3444	3.570(0.722-17.650)	0.1186	1.935(0.112-33.299)	0.6494	1.454(0.107-19.777)	0.7785
	Age		1.025(0.997-1.054)	0.0774	1.022(0.989-1.056)	0.1954	1.020(0.986-1.055)	0.2493	1.007(0.968-1.048)	0.7348

HR, hazard ratio; CI, confidence interval; BCS, breast conserving surgery; DM, diabetes; HTN, hypertension; COPD, chronic obstructive pulmonary disease; CKD, chronic kidney disease

Yellow box highlights the p-value less than 0.05, considered significant.
