# Supplementary figures and images for: Risk of Secondary Cancer after Adjuvant Tamoxifen Treatment for Ductal Carcinoma In Situ: A Nationwide Cohort Study in South Korea
Source: Diagnostics (Basel). 2023 Feb 20;13(4):792. doi: 10.3390/diagnostics13040792 (PMC9954831; doi:10.3390/diagnostics13040792)

### a. Multivariate analysis before matching

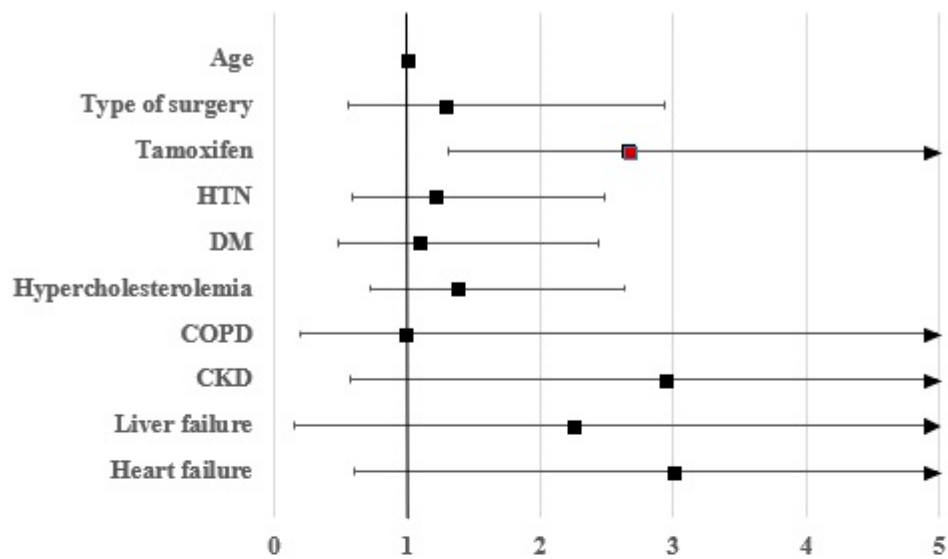

### b. Multivariate analysis after matching

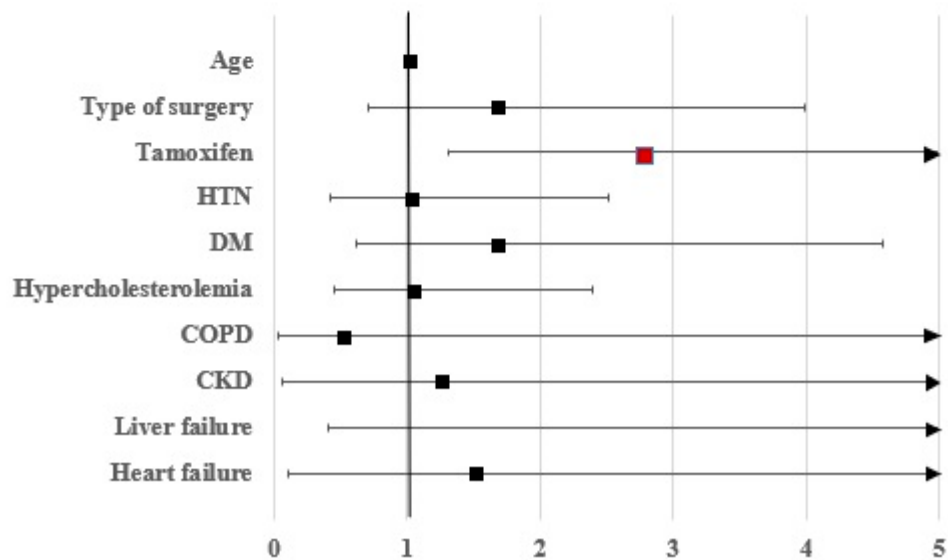

Supplement: Supplementary file 1 [file diagnostics-13-00792-s001.zip › diagnostics-2143508-Figure S1.pdf]
